# Exploring Harassment Directed Towards Employees on Social Media: A Scoping Review

**DOI:** 10.3390/bs16050797

**Published:** 2026-05-16

**Authors:** Samuel Farley, Molly Russell, Sarah Brooks, Iain Coyne

**Affiliations:** 1 University of Sheffield Management School, University of Sheffield, Sheffield S10 2TN, UK; s.j.farley@sheffield.ac.uk (S.F.); s.brooks@sheffield.ac.uk (S.B.); 2 Students Organising for Sustainability, Stockport SK1 1PN, UK; 3 Loughborough Business School, Loughborough University, Loughborough LE11 3TU, UK

**Keywords:** social media, harassment, sexual harassment, cyberbullying, online hate, scoping review

## Abstract

Recent reports indicate that employees are being subjected to harassment on social media. However, research in this area is highly interdisciplinary, often existing in disciplines that cater to occupational groups, such as politicians, journalists, or education professionals. We therefore conducted a scoping review to synthesize research in this area. Our scoping review sought to identify (1) the nature of social media harassment towards employees, (2) specific risk factors, and (3) how organizations manage the problem. We conducted searches of the Web of Science and Scopus databases, alongside keyword searches of Google Scholar. Our approach aligned with PRISMA recommendations for conducting scoping reviews, and the searches produced 47 studies, of which 35 met the inclusion criteria. Analyses revealed the varied nature of social media harassment towards employees, reflected in the use of 14 different labels to describe social media harassment. Only five studies addressed risk factors for experiencing harassment, which included greater prominence and visibility, more active use of social media, and working in an organization where offline harassment occurs. Moreover, just six studies have examined organizational responses to the problem, and were largely seen as ineffective, although thirteen studies addressed how individuals coped with social media harassment. This is the first paper that reviews research on social media harassment directed at employees. To consolidate this research area, we offer suggestions aimed at reducing construct proliferation and promoting a more coherent research agenda.

## 1. Introduction

Over the past 10 years, it has become common for employees to communicate with their colleagues and members of the public through social media accounts. According to reports, more than 60% of the world’s population uses social media (Kemp, 2022) and two-thirds of working US adults on Facebook have ‘friend connections’ with their work colleagues (Rothbard et al., 2022). Whilst social media has produced various benefits for employees, such as the ability to engage in new forms of voice behavior (Behrend et al., 2024), a slew of news reports have highlighted how it is used to harass workers. This harassment can be enacted by strangers; for example, climate and vaccine scientists have been abused by members of the public who disagree with the conclusions of their work (Nogrady, 2024). It can also be conducted by one’s colleagues, as members of the British Armed Forces have subjected female colleagues to unsolicited out-of-work messages, unwanted contact, and invitations to have sex (Guardian, 2023).

Given the widespread use of social media, there have been calls for more research on its use as a platform to harass workers (Behrend et al., 2024). Yet whilst research has started to examine how different working populations experience and cope with social media harassment (Celuch et al., 2022; Oksanen et al., 2020), this research has been published across a wide range of disciplinary boundaries, including communication science, computing, politics, journalism studies, and management. Researching a phenomenon from different disciplinary perspectives can provide a range of methodological and theoretical insights. However, unless evidence from diverse disciplines is synthesized, there is a risk that different terminology may be applied to the same concepts. This risks construct proliferation, which occurs when “research streams are built around ostensibly new constructs that are theoretically or empirically indistinguishable from existing constructs” (Shaffer et al., 2016, p. 81). The danger of construct proliferation is that the field becomes fragmented, with the development of overlapping constructs that examine the same relationships (Hershcovis, 2011). Research may also progress at a slower pace and in a less coherent manner, as the same research questions may be addressed in different disciplines, which promotes redundancy rather than new contributions to knowledge.

Our study therefore seeks to provide a holistic view of existing research on employee experiences of social media harassment through a scoping review that summarizes research findings to date. In summarizing the literature, we make three main contributions. First, we outline the key constructs of focus and illustrate the nature of social media harassment for those who experience it. Second, we examine existing research on risk factors. Third, we consider how organizations are responding to social media harassment directed at employees.

### 1.1. Social Media Harassment

Social media has been defined as “digital platforms that facilitate information sharing, user-created content, and collaboration across people” (McFarland & Ployhart, 2015, p. 1653). This broad definition encompasses the variety of platforms that employees may use to connect with one another and the wider public, such as networking sites (e.g., Facebook, LinkedIn, Twitter/X), video- and picture-sharing platforms (e.g., Instagram, YouTube), and instant messaging services (e.g., SMS, WhatsApp) (Behrend et al., 2024). Whilst organizations may have their own platforms that align with this definition of social media, much of the research to date has been conducted on public social media platforms (e.g., Facebook; Twitter/X). This may be because organizational actors would face sanctions for overtly harassing a colleague on an organizational platform (Farley et al., 2021).

Researchers have highlighted how several features of social media can alter the likelihood that harassment will occur, as well as how it is experienced for the parties involved (Celuch et al., 2022; Van Laer, 2014). Notably, anonymity can be readily achieved in social media by using fake accounts and bots (Orben et al., 2024), which can offer protection to perpetrators, potentially increasing the likelihood of harassment. Social media can also increase the availability of targets, which refers to how easily they can be contacted (Orben et al., 2024). This allows perpetrators greater reach and access to targets. Other notable features include heightened visibility (the extent to which messages are viewable by others), replicability (the extent to which messages can be duplicated and shared with others), persistence (the extent to which messages endure in their original form), and bandwidth (the breadth of socio-emotional cues that messages on social media transmit) (Orben et al., 2024). Whilst it is important to note that reviews of online harassment constructs have been undertaken in previous research (Saha et al., 2024; Shahwar & Dhar, 2024), these reviews were not domain specific. That is, they focused on forms of harassment that could be enacted over differing media channels, such as email, video conferencing, and instant messaging. Our review focuses solely on harassment conducted via social media, as the bounded nature of social media may permit clearer theoretical development and greater predictive power in subsequent research (Alvesson & Blom, 2022). Indeed, the unbounded, public nature of many social media platforms means that the actors involved may represent an entirely different population to those who engage in cyber harassment that takes place over organizational platforms such as email. For example, a politician may not be subjected to harassment over organizational forms of communication, but given their public role, they may be targeted much more readily on social media platforms by members of the public.

### 1.2. Review Aims

Our review has three aims. First, we seek to understand the nature of employee-based social media harassment by examining the main harassment constructs and behaviors that have been examined in research to date. Research on employee experiences of social media harassment has examined a wide range of behaviors, including sexual harassment (Scarduzio et al., 2021), hate speech (Oksanen et al., 2022), bullying (Mandalaki & Pérezts, 2023) and trolling (Akhtar & Morrison, 2019). However, for the purposes of this review, we will use the term harassment in a broad manner to encompass all forms of interpersonal mistreatment that an individual or group of individuals use that may cause distress (Van Laer, 2014).

When research is conducted across disciplines, different labels may be applied to constructs. Yet, even when the terminology applied is consistent, harassment is an umbrella term that covers a range of constructs that may differ in nature, such as racism, sexual harassment, and trolling (Hershcovis, 2011). It is therefore important to establish whether specific forms of harassment are over- or underrepresented in the research to date so that research efforts can target under-researched phenomena. In addition, it is important to review whether meaningful differences exist between harassment constructs or whether construct proliferation is present, which could obscure research progress. Finally, by reviewing the behaviors involved and the wider context surrounding these behaviors, our review illustrates what social media harassment is like for employees who experience it.

Second, we wanted to establish the risk factors for experiencing social media harassment. Given the reach and access provided by social media, much of the early research has seemed to focus on working populations who have a visible presence and who engage in communication on these platforms as part of their job role, such as politicians (Ward & McLoughlin, 2020), journalists (Tandoc et al., 2023), and academics (Oksanen et al., 2022). Workers in these professions are often targeted by strangers and members of the public, who would be classed as organizational outsiders. Organizational outsiders can also include clients, customers, members of the public, and service users (e.g., patients, pupils). As such, social media harassment has also been reported among those who work with service users, such as social workers, teachers, and health professionals. Social media harassment can also be enacted by organizational insiders, such as leaders and colleagues. Yet research suggests that cyberaggression from organizational insiders has a different range of antecedents to that enacted by outsiders (Farley et al., 2024). Therefore, one aim of this review is to develop an understanding of some of the risk factors that enhance the likelihood of social media harassment from both organizational insiders and outsiders. This is important to establish, as preventative measures cannot be fully developed without an understanding of which populations are most regularly targeted and why.

A final aim of the review is to develop an understanding of how organizations can prevent and address social media harassment towards employees. Developing this understanding is important because organizations may not know how to appropriately manage an employee who has enacted harassment over social media (Mainiero & Jones, 2013). Organizations may have to tailor their approach according to whether the abuse has come from organizational insiders or organizational outsiders. Nevertheless, organizations may not be doing enough to support those who are affected (Nogrady, 2024), which may be because confusion exists about who should be responsible for addressing the issue, with leadership, human resources, and security all having a role to play. Given that research on social media harassment of employees has only developed in recent years, we wanted to determine how organizations manage the issue and whether there is any evidence of its efficacy.

Our review questions are as follows:

1. *What is the nature of employee-based social media harassment?*
2. *What are the risk factors for experiencing employee-based social media harassment?*
3. *How do organizations manage employee-based social media harassment?*

## 2. Method

We conducted a review, following the PRISMA extension for scoping reviews checklist, to address our stated research questions (please see Supplementary Materials). We pre-registered the review on the Open Science Framework (OSF) on 8 April 2024, which detailed our research questions, search strategy, inclusion/exclusion criteria, and screening/extraction approaches (our OSF registration can be viewed at the following link: https://osf.io/4ahuj/overview?view_only=2ed450c05d07403d937b068b52a1a536, accessed on 8 April 2026).

### 2.1. Literature Search

As research on the harassment of employees over social media has evolved in different disciplines, we adopted a broad and inclusive approach to the development of our search protocol (Tranfield et al., 2003). We first specified terms relating to social media and personal messaging: *social media, social network, online platforms, personal messaging, WhatsApp, instant messaging,* and *social media platform.* We then used the ‘AND’ function to include the following keywords on employees: *worker, employee, staff, organization,* and *organisation.* Finally, we used the AND function to add a string of terms relating to harassment: *bullying, sexting, aggression, sexual harassment, abuse, trolling, cyberbullying,* and *aggression*. This search protocol was initially used to search the Web of Science database. However, to increase the inclusiveness of the search, we replicated this search strategy using the SCOPUS database. When conducting the searches in both databases, we used the filter functions to only include papers published in English, in academic journals, and after the year 2000. The searches produced 2736 papers in the Web of Science database and 1472 papers in the Scopus database, which were all screened for inclusion.

**Figure 1:**
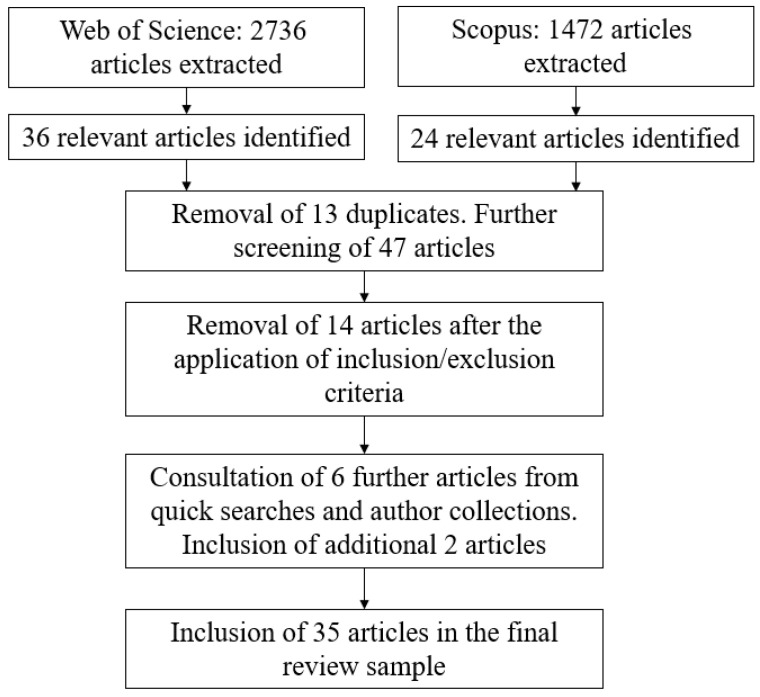
Diagram of the article selection process.

When screening papers in the two databases, we examined the title and abstract to determine whether the publication was relevant to our review questions. Most papers were excluded after reading the title and abstract, as the searches returned a high volume of papers that were clearly not relevant to our research questions. For example, theoretical papers and reviews were excluded once this was stated in the abstract, as were empirical papers that examined a non-work sample, such as children. If it was not clear from the title and abstract whether the paper met the inclusion criteria, the article was downloaded and read in full to identify whether it could be included in the review. All searches were concluded by 22 October 2024, and they were undertaken by the first and second authors. We also searched for relevant papers by consulting our own paper collections and by conducting a keyword search on Google Scholar to further increase the inclusivity of the search, which produced two additional articles (a total of six articles were screened from these searches, comprising 5 from Google Scholar and 1 from our own records). Figure 1 outlines our approach to the selection of papers included in the review.

### 2.2. Inclusion and Exclusion Criteria

We developed five criteria to ensure the selection of relevant papers. First, we were only interested in empirical, peer-reviewed papers that included an employee sample. Therefore, studies involving child, student^1^, and general population samples were excluded, as were review papers, chapters, and studies from ‘grey’ literature. Whilst studies needed to include an employee sample, we took an inclusive approach by including all studies that generated empirical data (qualitative or quantitative) and from a range of different sources (e.g., employee interviews, social media posts). Second, we did not include papers that used machine learning approaches to detect harassment on social media, as these were not relevant to our research questions. Third, we only included papers that examined harassment that explicitly took place on social media (e.g., Facebook, Instagram, WhatsApp). Therefore, studies examining general forms of online harassment where the medium used to channel the harassment was unspecified were not included in the review, nor were papers that referred to harassment over mediums such as email or video conferencing. In practice, we found that several papers (e.g., Forssell et al., 2024) addressed online harassment over more than one communications medium, such as social media and email. When this occurred, we read the paper in full to determine whether there were clear aspects of the analysis and interpretations that explicitly referenced social media. If clear and distinguishable aspects of the paper discussed social media, we included the paper in the review. Fourth, we only included papers published in English. Fifth, we excluded all papers published prior to the year 2000, as social media did not become widely used in general populations until the early 2000s.

To ensure the consistency of studies included in the review, the authors jointly discussed the inclusion and exclusion criteria applied to 53 articles that were considered relevant to the study research questions (47 articles were from database searches and 6 were from the additional searches). This process resulted in the retention of 35 articles that addressed social media harassment in the workplace. As we aimed to be inclusive in our retention of articles and given the wide variety of research methods and interdisciplinary backgrounds, we did not conduct a quality assessment of the articles included. This is common practice when reviews only include peer-reviewed sources (Mishra et al., 2021; Vasist & Krishnan, 2025). Furthermore, given the wide range of methodological approaches used in the included studies, it would not have been possible to develop a quality assessment process that could be standardized across the differing data collection and analysis approaches used in the included studies.

Following the inclusion of articles in the review, a coding process was conducted in stages. First, the second author coded the findings from all 35 articles to identify common themes related to the scoping review research questions. We adopted a descriptive approach to summarizing the results of the scoping review (Arksey & O’Malley, 2005). Specifically, we adopted a theoretical approach to coding, whereby papers were coded according to our research questions (Braun & Clarke, 2006). In practice, this involved organizing the papers in a spreadsheet and noting down how each paper addressed the research question. Second, a consistency check was conducted, whereby the first, third and fourth authors divided up each of the 35 studies to review the initial coding conducted by the second author. The research team then met online to resolve any disagreements in how the findings had been interpreted. Third, the first author reviewed how each study had been coded to organize how the research questions had been addressed, to ensure that the findings could be summarized consistently. This process revealed that 20 of the included studies provided information that was relevant to answering research question one, 5 were relevant to answering research question two, and 17 were relevant to answering research question three. Each study was relevant to at least one of the research questions, but some addressed more than one question.

## 3. Results

### Descriptive Characteristics of Included Studies

We extracted data from the final set of studies using a spreadsheet that captured information on each study’s harassment construct, geographic location, organizational context, methodology, sample, theoretical framing, and the research field of each study’s journal. Table 1 highlights key study information, but the spreadsheet can be viewed at the following OSF project: https://osf.io/tw54z/?view_only=2c374d394a434fbfad8022e3700420d6 (accessed on 8 April 2026). We also coded whether the study mentioned a specific social media website (e.g., Facebook) and whether the harassment was enacted by organizational insiders or outsiders.

The studies were published between 2016 and 2024, which highlights the nascent nature of the research field. A range of harassment constructs were investigated, with the most common being ‘online harassment’ (examined in 10 studies). Other constructs included ‘cyberbullying’ (5 studies), ‘online abuse’ (4), ‘sexual harassment’ (3), ‘trolling’ (2), ‘online hate’ (2), and ‘online incivility’ (2). The constructs of ‘abusive metajournalistic discourse’, ‘cyber mistreatment’, ‘deviant workplace behavior’, ‘maladaptive parasocial interaction’, ‘online academic bullying’, ‘online racism’ and ‘technology facilitated violence and abuse’ were each examined in just one study.

Formal construct definitions were not provided in every paper, but we recorded the definitions that were outlined in Table 2. This table shows that although construct labels (e.g., cyber mistreatment, technology-mediated violence and abuse, online hate) are often different, much of the content in the definitions appears similar. For example, Forssell et al. (2024), Gosse et al. (2024), and Celuch et al. (2022) each refer to ‘negative’, ‘harmful and disruptive’, or ‘violent’ behaviors occurring online. Some features did serve to differentiate definitions, as characteristics of intent and repetition were often mentioned in relation to cyberbullying/online bullying (La Regina et al., 2021; Noakes & Noakes, 2021; Smith et al., 2008). Furthermore, differing forms of abuse were also distinguishable, with sexual harassment investigated in three studies and racial harassment investigated in one study. Finally, whilst many of the definitions stated that the behavior is enacted online or over a particular communication medium, others were more or less specific in prescribing where the harassment takes place. For example, Forssell et al. (2024) highlighted that cyber mistreatment takes place via email and social media, whereas others do not mention the platform or online space whatsoever (Nayak et al., 2022; Ward & McLoughlin, 2020).

Most studies (22; 62.9%) adopted an inductive approach, which is perhaps unsurprising as social media harassment is a novel phenomenon. The other 13 studies used different theories to frame their data collection and analysis, including claims-making theory (Spector & Kitsuse, 1977) social identity theory (Tajfel & Turner, 1979), monstrification (J. J. Cohen, 1996), transactional stress theory (Lazarus & Folkman, 1984), and network analysis theory (Burkhardt & Brass, 1990). The only theory used in more than one study was routine activity theory (RAT) (L. E. Cohen & Felson, 1979), which was adopted in two studies. RAT argues that anti-social behavior is more likely to occur when a motivated offender encounters a suitable target (an individual who is accessible, of lower or equal status, and less likely to fight back), under conditions where a capable guardian is absent. The theory is highly applicable in the context of social media harassment, as a high volume of suitable targets are accessible on social media, where there is often little guardianship to police or prevent mistreatment.

The studies were primarily published in western countries (68.57% of studies were published in the west), including the UK (5 studies), USA (5), Finland (4), Canada (3), Denmark (1), France (1), Ireland (1), Israel (1), Italy (1), New Zealand (1), and Sweden (1). In contrast, 22.95% of studies were published from the Global South, from India (3), Nepal (2), Brazil (1), the Philippines (1), and South Africa (1). Three studies (8.57%) had a global focus that was not restricted to any single country. To better understand the research disciplines represented by the 35 studies, we coded them according to how their journals were described on each journal’s website (see Table 3). This showed the highly interdisciplinary nature of the topic, as the journals represented 12 different disciplines. The most commonly represented discipline was ‘computing and information’, with 10 studies (28.57%) being published in these journals. In contrast, 6 were published in communication journals, 4 were published in journalism studies journals, and 3 studies each were published in education, health and social care, and management journals. The other disciplines, each represented by a single study, were bullying, law, psychiatry, science, sexuality, and sport.

The organizational sectors represented included education (8 studies), politics (6), journalism (5), sport (3), and social work (2). Six studies focused on workers from a variety of organizations, although these samples often included sub-samples from the political or education sectors (Celuch et al., 2022; Celuch et al., 2024). The IT, healthcare, charity, and trade union contexts were each represented by a single study.

A range of data collection methods were used across the studies, including interviews, surveys, and text-based content analysis (see Table 1). Most studies (24; 68.6%) collected data directly from human employees via self-report methods (e.g., interviews, surveys). In contrast, nine (25.7%) utilized text-based methods to analyze data such as Facebook posts, tweets, and legal verdicts (two studies collected data from humans and text-based content). Only eight studies (22.9%) used a methodology that could be described as quantitative. These studies all used an online survey to collect data from employees, with inferential statistics being used to analyze the data in seven of the eight studies. The other 27 studies (77.1%) used methodologies that could be described as qualitative^2^, including interviews (13 studies), content analysis (9), autoethnographies (2), qualitative online surveys (2), case study analysis (1), observation (1), netography (1), process study (1), and sentiment analysis (1).

We attempted to code the studies according to whether they focused on harassment enacted by organizational insiders or outsiders. However, this proved challenging, as eight studies did not include information on the perpetrator’s identity, or they examined harassment from both insiders and outsiders. Nevertheless, 21 studies (60%) appeared to focus solely on outsider-enacted harassment, whereas just 6 (17.1%) focused on harassment from colleagues. Similarly, it was difficult to isolate the different social media platforms examined across the studies, as many did not state the specific platform, or examined more than one platform. Nonetheless, Facebook and Twitter/X were the most referenced platforms, with others including WhatsApp, Skype, Instagram, LinkedIn, and YouTube.

## 4. Results by Research Question

### 4.1. Research Question 1: What Is the Nature of Employee-Based Social Media Harassment?

Whilst each paper reviewed investigated a particular harassment construct, such as cyberbullying or online harassment, 20 studies (57%) went into greater depth by exploring how behaviors are experienced, as well as different methods of categorization. Specific harassment behaviors were highlighted across the studies, including sending offensive, angry, and inflammatory messages (Akhtar & Morrison, 2019; Oksanen et al., 2022), belittling and insolence (Rajbhandari & Rana, 2022), false information spreading (Forssell et al., 2024), cyber-stalking (Burns et al., 2024), privacy violations (Tenório & Bjørn, 2019) blaming/scapegoating (Nayak et al., 2022), criticism of work efforts (La Regina et al., 2021), using extreme language (Kagan et al., 2018), making threats (Barlow & Awan, 2016) and name-calling (Southern & Harmer, 2021). Some studies sought to categorize specific behaviors into broader categories. For example, Sanderson et al. (2020) analyzed tweets directed towards an athlete on Twitter/X, finding that they could be categorized into criticism, threats, and expressions of anger. In contrast, Southern and Harmer (2021) utilized Papacharissi’s (2004) civility typology to categorize harassment towards politicians as stereotyping, name-calling, labelling the recipient a liar, labelling them unintelligent, or profanity.

Other studies sought to categorize social media harassment by the subject or content of the message, rather than behaviorally. For example, Meggs and Ahmed (2024) categorized abusive tweets directed at athletes on Twitter/X as: (1) abuse without context (e.g., “*You are a loser*”); (2) abuse directed at sporting ability (e.g., “*Lazy and overrated*”); and (3) abuse directed at personal life (e.g., *“@userhandle is a wife beater*”). Adams et al. (2022) examined three types of online sexual harassment that differ in nature: (1) gender harassment (“*unwelcome verbal and nonverbal behaviors that are insulting, hostile, or degrading towards one’s gender*”; (p. 1842); (2) unwanted sexual attention (messages expressing sexual desires or intentions); and (3) sexual coercion (soliciting sexual content in return for rewards or benefits). Finally, Ward and McLoughlin (2020) distinguished between generic abusive content and hate speech, with the latter representing content that either threatens the target or refers to their demographic identity, such as their gender, ethnicity, or religion.

In many studies, the behaviors experienced were intrinsically linked to the target’s demographic characteristics. This was most notably the case for gender, with females experiencing sexualized or gendered abuse online (Akhtar & Morrison, 2019; Barlow & Awan, 2016; Every-Palmer et al., 2024; Mandalaki & Pérezts, 2023; Koirala, 2019; Tandoc et al., 2023), often in greater volume than males (Akhtar & Morrison, 2019; Every-Palmer et al., 2024). Specific behaviors include being sent unsolicited nude photos (Koirala, 2019), rape threats (Akhtar & Morrison, 2019), and gendered insults, such as ‘slag’ and ‘bitch’ (Barlow & Awan, 2016). It was noted that gendered and racial abuse was often sent reactively by perpetrators, often being triggered when targets speak out against gendered or racial injustices (Barlow & Awan, 2016) or take divisive public positions or actions (Akhtar & Morrison, 2019).

Several studies explored the broader context of harassment behaviors. There is a consensus that the nature of social media harassment often involves the repeated and strategic use of public or private spheres (Kagan et al., 2018; Tandoc et al., 2023). For example, politicians and others in publicly visible roles may be disproportionately targeted by social media harassment due to the visibility of their position (Akhtar & Morrison, 2019). The mobilization of these public spaces can serve as a tactic to garner public support, amplifying the impact on the target’s reputation, or that of their organization (Forssell et al., 2024; Kagan et al., 2018). In some cases, social media harassment begins systemically, with public critique by an influential figure leading to substantial hostility (Tandoc et al., 2023).

Several studies suggest that the nature of social media harassment is excessive, repetitive, and frequent. This may manifest in sustained behaviors, such as excessive criticism (Oksanen et al., 2022), occur on multiple platforms (Every-Palmer et al., 2024), and be exacerbated by sociopolitical events, such as COVID-19 (Every-Palmer et al., 2024). Indeed, Holton et al. (2021) sought to develop distinct behavioral categories to reflect patterns of escalation, referring to the progression in intensity and severity over time. Categories included *acute harassment* (less personal harassment focused on a single event or topic), *chronic harassment* (sustained harassment over time from one or multiple users), and *escalatory harassment* (direct threats of a professional, personal, or organizational nature).

### 4.2. Research Question 2: What Are the Risk Factors for Experiencing Employee-Based Social Media Harassment?

It was common for studies to speculate on possible harassment risk factors. For example, it was noted that speaking out about a divisive societal issue, greater public visibility, and being targeted by an influential figure could cause social media harassment. However, only five (14%) studies empirically examined risk factors of social media harassment. Therefore, the conclusions of these studies should be treated as preliminary and limited, rather than well established. Farrell et al. (2020) conducted a study of the tweets from UK Members of Parliament (MPs) that attract the most abuse, finding that communicating about divisive topics attracts abusive replies, alongside personal characteristics of the MP (e.g., MP prominence, gender, political party, and ethnicity). Indeed, the authors noted that when female MPs with ethnic minority backgrounds post about inequality, it tends to attract more abusive responses. However, Al-Rawi et al. (2024) found no gender differences among journalists most targeted by social media. Oksanen et al. (2020) indicated that those targeted by cyberbullying to a greater extent were younger male employees, who were active users of professional social media, and involved in social media identity bubbles, which are identity driven online cliques based upon a need to belong to a social group (Latikka et al., 2022).

Herovic et al. (2019) suggested that organizational culture influences sexual harassment experiences, specifically the spillover between the targets’ online and offline spaces, which could be considered antecedents of online sexual harassment. Therefore, where sexual harassment is a common feature of face-to-face interactions within a workplace, it may be more likely to spillover and occur in social media interactions.

Finally, Pasquier et al. (2024) analyzed a Facebook union group to outline the processes that lead members to become trolls. They suggested that the three effects of discording, disordering, and disgusting enable the ‘monstrification’ process through which people become trolls. Discording involves the misalignment or disharmony between online and offline democratic processes. Disordering refers to the lack of structures online that allow for democratic discussion, thereby allowing chaotic and unchallenged interactions. Finally, disgusting referred to the mutual perceptions of moral deviance that emerged between union leaders and members.

### 4.3. Research Question 3: How Do Organizations Manage Employee-Based Social Media Harassment?

The organizational management of social media harassment is dependent on the extent to which the organization is aware of the situation and the extent to which employees desire or require assistance with it. Therefore, when attempting to answer this question, we included studies that focused on how individuals manage the situation, as well as how organizations respond. These were reviewed in two separate sub-themes. A total of 17 studies (48.6%) were reviewed to answer this research question.

### 4.4. Sub Theme 1: Individual Management of Social Media Harassment

This sub-theme focuses on the strategies targets employ to manage social media harassment, which were referenced in 13 (37%) of the 35 papers. The strategies included self-protection, resistance, acceptance and self-blame (Veletsianos et al., 2018), ignoring the harassment entirely (Holton et al., 2021; Rajbhandari & Rana, 2022; Tandoc et al., 2023), avoiding certain platforms (Koirala, 2019), developing a ‘thick skin’ (Al-Rawi et al., 2024; Koirala, 2019), increasing self-efficacy to overcome harassment (Rajbhandari & Rana, 2022), self-censorship (Al-Rawi et al., 2024; Celuch et al., 2022), and reporting the harassment (Al-Rawi et al., 2024; Noakes & Noakes, 2021; Scarduzio et al., 2021). A few studies discussed both functional and dysfunctional management strategies, including exercise, unhealthy eating, and consuming alcohol (Bhat, 2024; Holton et al., 2021).

The identities of targets may influence individual management strategies. Several studies noted that individual management strategies were often gendered (Al-Rawi et al., 2024), with males more likely to respond to messages of harassment (Akhtar & Morrison, 2019) and females more likely to self-censor (Celuch et al., 2024). Moreover, being a more active user of social media was linked to using active strategies aimed at attempting to stop the perpetrator by reacting to the message (Celuch et al., 2022), as opposed to adopting a more passive strategy.

Sharing experiences of social media harassment also emerged as a prevalent individual management strategy. Individuals often turned to friends, family, and colleagues for emotional, psychological (Rajbhandari & Rana, 2022), and logistical support (Tandoc et al., 2023). This can help mitigate the use of so-called ‘negative’ coping strategies, such as self-censorship (Celuch et al., 2024). Additionally, forming supportive networks outside of one’s organization was reported as beneficial (Bhat, 2024), such as those involving messages of solidarity from bystanders (Tandoc et al., 2023). Scarduzio et al. (2021) explored why targets of social media harassment report online harassment to their organization. While some report harassment to protect others and/or believe that effective support will follow, others refrain from reporting to maintain relationships at work and downplay the harassment’s severity.

### 4.5. Sub Theme 2: Organizational Management of Social Media Harassment

Six (17%) of the thirty-five studies included content on the management of employee-based social media harassment by organizations. Organizational management can involve primary, secondary, or tertiary actions to prevent harassment (Hershcovis et al., 2015), whereby primary actions are preventative measures, secondary actions seek to deal with harassment when it occurs, and tertiary actions involve supporting the target after harassment has occurred.

Two studies seemed to examine secondary interventions that can be used to deal with social media harassment. Dineva and Breitsohl (2022) identified six trolling management strategies for organizations. Three strategies were considered ‘direct’: expurgating (removing trolling comments when requested by other users), asserting (posting a mission-related comment with no explanation or justification) and mobilizing (making an appeal during a trolling incident for users to change their behavior). In contrast, the other three were considered ‘indirect’: non-engaging (not taking any action to manage trolling), educating (providing educational information about an ethical issue causing the trolling), and bolstering (affirming a supporter’s comment in a trolling incident). Overall, the direct strategies were considered more efficacious than the indirect ones, based upon the frequency of subsequent trolling comments. Tenove et al. (2023) examined how political campaign teams dealt with social media harassment by evaluating the content according to whether it threatened either: (1) security and psychological wellbeing; (2) strategic campaign activities; and (3) inclusive democratic discourse. Action was then taken based upon how the threat was categorized, including reporting or blocking users, ignoring and muting accounts, and monitoring/blocking repeated harassment which may be attributable to ‘bots’. However, the impact of these activities was not reviewed in the study.

Ineffective organizational management was raised as an issue in four studies. Kilvington and Price (2019) highlighted ineffective management in responding to racism on social media by both football clubs and professional bodies, which was attributed to a culture of secrecy, inconsistent approaches, and a lack of clear policies. Similar themes were apparent in a study on how Canadian colleges and universities protect staff from ‘technology-facilitated violence and abuse’ (TFVA) (Gosse et al., 2024). Whilst policies exist to deal with this issue, their coverage was often limited by focusing on work communications and spaces and omitting non-work communications.

When investigating how news organizations respond to their journalists’ complaints of harassment, Bhat (2024) described the actions taken as “palliative measures” (p. 343) which frame social media harassment as the responsibility of the individual target. This theme was also apparent in Holton et al.’s (2021) study on journalists, with American news workers feeling a lack of tangible support. Organizational recommendations included ignoring the perpetrator, reporting the harassment on the social media platform, and exercise or meditation (Holton et al., 2021).

Whilst organizations may view social media harassment as an individual problem, they may also be unprepared to deal with the issue due to a host of other factors. These include prioritizing physical safety over digital safety, ineffective reporting processes that do not align with the speed and frequency of harassment (Gosse et al., 2024), confusion amongst organizations regarding what work is currently being done to manage social media harassment, and a shortage of resources (Kilvington & Price, 2019).

## 5. Discussion

This scoping review sought to answer three research questions. The first question was to determine the nature of employee-based social media harassment. Our findings revealed that 14 different labels have been used to describe harassment constructs (e.g., cyber mistreatment, online sexual harassment, online harassment, trolling) with labels often broken down into further subcategories of harassment. Whilst multiple harassment labels exist, definitions of several harassment constructs appear to be conceptually similar, with a typical definition referring to ‘negative’, ‘violent’, or ‘abusive’ behaviors that occur online. Where differences did exist, they referred to the nature of the behavior (whether it was intentional, repeated, sexual, or racial) and the channel through which it was mediated (e.g., the internet, social media, digital technologies). The work-related nature of harassment was only mentioned in two definitions (Noakes & Noakes, 2021; Robinson & Bennett, 1995) and definitions did not differentiate between perpetrators as either organizational insiders or outsiders.

A key question moving forward is whether a unifying definition would aid research on employee-based social media harassment. The presence of so many differing definitions suggests the field is suffering from construct proliferation. This is problematic, as construct proliferation obscures theoretical development, impedes the creation of new knowledge, prevents collaboration between researchers and practitioners, and reduces the influence of scientific disciplines (Shaffer et al., 2016). In her seminal article on how to address the construct proliferation of offline workplace aggression constructs, Hershcovis (2011) argued that the field would benefit from agreeing on a single broad operational label. Any proposed differences between constructs could then be examined as moderators. Applying this suggestion to social media harassment research would require the adoption of a single label and definition. Researchers could then empirically examine how separate features of harassment, such as intent, repetition, form (e.g., sexual, racial harassment), social media site, and perpetrator status, differentially affect the experience for the actors involved. With this in mind, a simple broad definition of employee-based social media harassment could be ‘expressions of harassment directed towards employees on social media channels.’

Our second review question sought to identify risk factors for experiencing social media harassment from organizational insiders and outsiders. Only five studies had examined possible predictors of social media harassment, which means that extensive further research is needed to better establish the risk factors involved. The preliminary research suggests that individuals are more likely to be targeted when they are more prominent or well-known (Farrell et al., 2020) and when they are more active users of social media (Oksanen et al., 2020). Connected to this is the notion that harassment occurs when individuals post ‘politicized’ content (Akhtar & Morrison, 2019; Barlow & Awan, 2016), such as information on gun control, climate change, political decisions, and diversity, equity, and inclusion (DEI) (Al-Rawi et al., 2024; Farrell et al., 2020; Nogrady, 2024). In certain professions, most notably journalism and politics, it can be both beneficial and unavoidable to be well known and active on social media. However, this seems to come with the risk of experiencing social media harassment (Celuch et al., 2024).

Sociodemographic factors such as ethnicity, gender and age (Farrell et al., 2020; Oksanen et al., 2020) may influence the type and volume of harassment that targets experience, although the influence of gender is equivocal (Al-Rawi et al., 2024). Within cyberbullying literature, sociodemographic risk factors have included gender (Forssell, 2016), extent of social support (McCord et al., 2024; Muhonen et al., 2017) and type and frequency of online behavior (Kowalski et al., 2014). Therefore, future research should investigate further whether socio-demographic factors influence the nature and prevalence of social media harassment. In addition, it was suggested that social media harassment from insiders may occur when an offline disagreement spills over to the online domain, and vice versa (Herovic et al., 2019; Pasquier et al., 2024). It may also be the case that where harassment is normalized within face-to-face organizational interactions, it is more likely to occur on social media (Herovic et al., 2019). Previous work has found that role conflict, availability expectations, and poor-quality work are related to cyberaggression from insiders, but not outsiders (Farley et al., 2024). However, it was notable that very little research addressed predictors of social media harassment from organizational insiders, and this should be prioritized in future research.

Our final review question sought to identify how organizations manage employee-based social media harassment. In answering this question, we reviewed research on how individuals and organizations manage it. Considerably more research has focused on how individuals respond, which details strategies including self-censorship, confronting abusers, seeking support, and reporting it. Studies on organizational responses often concluded that the action taken by organizations was largely ineffective, as they tend to shift responsibility for resolution onto the target, as opposed to dealing with the matter directly (Bhat, 2024; Holton et al., 2021). As such, a rather dispiriting finding from our review is that very little empirical evidence exists that can be used by organizations to develop supportive measures.

### Theoretical Implications

Our study provides the ‘lay of land’ concerning existing research on social media mistreatment, and a notable finding was that most studies included in the review were inductive. Although this is not surprising since employee-based social media harassment is a relatively new phenomenon, it does highlight that theoretically informed studies could allow for fresh discoveries in this research area.

Routine activity theory (L. E. Cohen & Felson, 1979) was the only theoretical framework used in more than one of the studies included in the review. The theory states that anti-social behavior occurs when three elements are present: a motivated offender, a suitable target, and the absence of capable guardians. Motivated offenders are persons with the intention or ability to commit a crime, whereas suitable targets are those who are attractive to offenders and vulnerable to attack, with key characteristics encompassing value, visibility, accessibility and inertia (L. E. Cohen & Felson, 1979). Capable guardianship refers to actors who have the capacity to prevent a violation (L. E. Cohen & Felson, 1979).

Our findings shed light on the nature of anti-social behavior online, as well as characteristics of motivated offenders, suitable targets, and capable guardians. Viewing others as morally deviant was a risk factor for becoming a motivated offender (Pasquier et al., 2024). In contrast, risk factors for becoming a suitable target include having greater online prominence, actively using social media, posting about divisive topics, belonging to social media bubbles, and working in an organization where offline harassment occurs (Farrell et al., 2020; Oksanen et al., 2020). Examples of effective guardianship included campaign teams who blocked abusive users, muted abusive accounts, and monitoring harassment that might be attributable to a ‘bot’ (Tenove et al., 2023). Similarly, the strategies of removing trolling comments, appealing to trolls to change their behavior, and posting mission-related content in response to trolls were considered more effective than indirect trolling management strategies (Dineva & Breitsohl, 2022). However, there is a clear need for future research on the characteristics of offenders, targets, and guardians in relation to employee-based social media harassment.

## 6. Limitations and Future Directions

One potential limitation of our review was that we did not conduct a quality review, and thus the study findings were given equal weight, regardless of methodological rigor. Furthermore, the qualitative nature of most of the included studies affected how we sought to answer our research questions, as we were primarily able to answer the questions descriptively, as opposed to summarizing cause-and-effect relationships that are common in quantitative research. Indeed, one of the challenges inherent in mixed-methods reviews concerns how to integrate different types of data when answering a research question. Nevertheless, the advantage of our more inclusive approach was that we were able to develop an interdisciplinary and representative picture of employee-based social media harassment research. In addition, our mixed-methods review balances rich insights on the nature of social media harassment with how it relates to risk factors and management strategies. A second limitation is that we only included peer-reviewed studies that had been published in English, which increases the risk of publication bias.

Our findings suggest a few areas that require further research attention. First, most studies in this area take a ‘victim-centric’ approach, which means that our understanding of the perpetrators of social media harassment is underdeveloped. Therefore, steps should be taken to understand who is conducting this harassment and why. Research suggests that 20% of social media content comes from bots (Ng & Carley, 2025), which indicates that much of the harassment generated may not even be enacted by humans. Another promising avenue for future research would involve exploiting the nature of social media to better understand how episodes of harassment can be effectively de-escalated. As most social media content is in written form, researchers could conduct experimental studies to identify factors that contribute to harassment de-escalation. This type of research could also be conducted in field studies, as Brett et al. (2007) used analysis of eBay sales disputes to identify that cases were more likely to be settled when causal accounts were provided to give the other party context, alongside language that affirmed face. Third, as most studies have utilized an inductive approach, the field may benefit from more studies that use a deductive approach to produce generalizable findings. Quantitative studies were underrepresented in the cohort of included studies, but they may be useful in determining how differing antecedent and outcome variables relate to social media harassment. For example, researchers could use the online disinhibition effect (Suler, 2004) or the social identification/deindividuation (SIDE) model (Lea et al., 2001; Spears & Lea, 1994) to study how and why individuals are motivated to engage in harassment towards employees.

## 7. Conclusions

This study provides the lay of the land on employee-based social media harassment research. By synthesizing research from across disciplines, we shed light on construct proliferation within the research area and suggest a simple definition that can be used by researchers across disciplines to consistently investigate the phenomenon. We also provide insights into the research questions addressed thus far and the methodological and analytical approaches used to draw conclusions. Our findings suggest that most studies utilize a qualitative approach to provide insights into how the phenomenon is experienced by victims. Therefore, opportunities exist to explore risk factors and organizational responses in greater depth using deductive approaches. By doing so, researchers will be able to provide greater insights into how societies, organizations, and individuals can prevent and limit employee-based social media harassment. One practical implication arising from our review suggests that employees are more likely to be targeted when individuals are well known or post about controversial or politicized topics. Although organizations may not be able to prevent harassment towards these individuals, they can provide resources in the form of secondary actions that help targets to deal with the harassment. This could include public statements of support, training on how to manage threats of abuse, and the provision of additional security.

## Figures and Tables

**Table 1:** Summary of included studies.

Authors	Research Field	Harassment Construct	Geographic Location	Organizational Context	Sample	Methodology
Adams et al. (2022)	Sexuality	Sexual harassment	USA	Various	Employed students (*n* = 189)	Online survey (quantitative)
Akhtar and Morrison (2019)	Computing and Information	Trolling	UK	Politics	Member of parliament (*n* = 181)	Online survey (quantitative)
Al-Rawi et al. (2024)	Journalism	Abusive metajournalistic discourse	India (and global)	Journalism	Journalists (*n* = 12)	Content analysis and interviews
Barlow and Awan (2016)	Computing and Information	Online hate	Denmark	Education	Academic staff (*n* = 2)	Autoethnography
Bhat (2024)	Journalism	Online harassment	India	Journalism	Journalists (*n* = 24)	Interviews
Burns et al. (2024)	Health and Social Care	Online abuse and harassment	Ireland	Social work	Social workers (*n* = 83)	Online survey (quantitative and qualitative)
Celuch et al. (2024)	Computing and Information	Online harassment	Finland	Various	University employees (*n* = 2492); politicians (*n* = 510); media professionals (*n* = 695)	Online survey (quantitative)
Celuch et al. (2022)	Computing and Information	Online harassment	Finland	Various	University employees (*n* = 2492); politicians (*n* = 510)	Online survey (quantitative)
Dineva and Breitsohl (2022)	Computing and Information	Trolling	Global	Animal charity	Trolling comments (*n* = 29,929)	Netography
Every-Palmer et al. (2024)	Psychiatry	Online harassment	New Zealand	Politics	Member of parliament (*n* = 54)	Online survey (quantitative and qualitative)
Farrell et al. (2020)	Computing and Information	Online abuse	UK	Politics	Twitter/X corpus on members of parliament (*n* = 574)	Content analysis
Forssell et al. (2024)	Education	Cyber mistreatment	Sweden	Education	Teachers and principals (*n* = 31	Interviews
Gosse et al. (2024)	Education	Technology facilitated violence and abuse (TFVA)	Canada	Education	Workplace harassment policies (*n* = 129) and university employees (*n* = 10)	Content analysis and interviews
Herovic et al. (2019)	Communication	Sexual harassment	USA	Various	Employed students (*n* = 15)	Interviews
Holton et al. (2021)	Journalism	Online harassment	USA	Journalism	Journalists (*n* = 31)	Interviews
Kagan et al. (2018)	Health and Social Care	Cyberbullying	Israel	Social work	Facebook pages (*n* = 5), weblogs (*n* = 11) and YouTube channels (*n* = 2)	Content analysis
Kilvington and Price (2019)	Communication	Online racism	UK	Sport	Football charity workers, administrators and club employees (*n* = 6 and *n* = 7)	Interviews and online survey (qualitative)
Koirala (2019)	Communication	Online harassment	Nepal	Journalism	Female journalists (*n* = 48)	Interviews
La Regina et al. (2021)	Health and Social Care	Cyberbullying	Italy	Healthcare	Facebook posts (*n* = 217)	Content analysis
Mandalaki and Pérezts (2023)	Management	Cyberbullying	France	Education	Academic staff (*n* = 2)	Autoethnography
Meggs and Ahmed (2024)	Sport	Online abuse	Global	Sport	Tweets directed towards highly paid athletes (*n* = 10)	Sentiment analysis
Nayak et al. (2022)	Management	Online harassment	India	IT	HR practitioners (*n* = 26)	Interviews
Noakes and Noakes (2021)	Science	Online academic bullying	South Africa	Education	Emeritus Professor (*n* = 1)	Case study analysis
Oksanen et al. (2022)	Education	Online hate and harassment	Finland	Education	University employees (*n* = 2492)	Online survey (quantitative)
Oksanen et al. (2020)	Computing and Information	Cyberbullying	Finland	Various	Finnish employees (*n* = 563 and *n* = 1817)	Online survey (quantitative)
Pasquier et al. (2024)	Management	Trolling	Canada	Trade union	Interviews (*n* = 35), meetings (*n* = 15) and Facebook content (*n* = 326 and *n* = 3307)	Process study
Rajbhandari and Rana (2022)	Bullying	Cyberbullying	Nepal	Education	Teachers (*n* = 20)	Interviews and observation
Sanderson et al. (2020)	Communication	Maladaptive parasocial interaction	USA	Sport	Tweets (*n* = 512)	Content analysis
Scarduzio et al. (2021)	Communication	Sexual harassment	USA	Various	University employees (*n* = 213)	Online survey (qualitative)
Southern and Harmer (2021)	Computing and Information	Online incivility	UK	Politics	Tweets (*n* = 117,802) towards members of parliament (*n* = 500)	Content analysis
Tandoc et al. (2023)	Journalism	Online harassment	Philippines	Journalism	Female journalists (*n* = 8)	Interviews
Tenório and Bjørn (2019)	Computing and Information	Online Harassment	Brazil	Various	Legal verdicts on labor law violations (*n* = 106)	Content analysis
Tenove et al. (2023)	Communication	Online incivility	Canada	Politics	Candidates and campaign staff from the 2019 Canadian federal election (*n* = 31)	Interviews
Veletsianos et al. (2018)	Computing and Information	Online harassment	Global	Education	Female scholars (*n* = 14)	Interviews
Ward and McLoughlin (2020)	Law	Online abuse	UK	Politics	Tweets (*n* = 270,717) towards members of parliament (*n* = 573)	Content analysis

**Table 2:** Construct definitions.

Construct	Definition	Study
Cyberbullying	“An aggressive, intentional act carried out by a group or individual, by issuing electronic forms of contact repeatedly and over time against a victim who cannot easily defend him or herself” (Smith et al., 2008, p. 376).	Kagan et al. (2018)
Cyberbullying	“An intentional, aggressive act or acts over a period of time to inflict harm on the victim by utilizing various electronic forms of expression” (La Regina et al., 2021, p. 2).	La Regina et al. (2021)
Cyberbullying	“Experienced when using various social networking sites such as Facebook and Twitter for open communications and may be deliberately imposed on someone to harm or harass” (Rajbhandari & Rana, 2022, p. 95).	Rajbhandari and Rana (2022)
Cyber Mistreatment	“A non-physical negative behaviour, commonly involving written text, images or videos forwarded by email or on social media”. (Forssell et al., 2024, p. 2).	Forssell et al. (2024)
Deviant Workplace Behavior	“Voluntary behaviour that violates organisational norms and endangers the organisation’s well-being or its members or both” (Robinson & Bennett, 1995, p. 557).	Nayak et al. (2022)
Online Abuse	“Messages directed at a specific person with the intent to cause harm or distress” (Ward & McLoughlin, 2020, p. 55).	Ward and McLoughlin (2020)
Online Abuse & Harassment	“A set of behaviours and practices, whether single or multiple events, by individuals or groups using digital technologies and devices, social media platforms, and/or the Internet to send or post direct or implicit messages that are abusive, threatening, or stalking or harassing behaviours of a person” (Burns et al., 2024, p. 3).	Burns et al. (2024)
Online Academic Bullying (OAB)	“A drawn-out situation in which its recipient experiences critique online by employees in HE that is excessive, one-sided and located outside of typical scholarly debate and accepted standards for its field” (Noakes & Noakes, 2021 p. 1).	Noakes and Noakes (2021)
Online Harassment	“Abusive behaviours enabled by technology platforms used to target specific users” (Tenório & Bjørn, 2019, p. 296).	Tenório and Bjørn (2019)
Online Harassment	“A practice where an individual or group use the Internet to harass, harm, or ridicule another person using either a fake or real identity” (Koirala, 2019, p. 47).	Koirala (2019)
Online Harassment	“Online harassment (i.e., cyberharassment) encompasses a wide range of violent behaviors in the online space” (Celuch et al., 2022, p. 1).	Celuch et al. (2022)
Online Hate	“An expression that is abusive, insulting, intimidating, harassing, and incites violence or discrimination” (Barlow & Awan, 2016, p. 2).	Barlow and Awan (2016)
Online Hate	“Targeting either individuals or groups of people with intensive and hostile statements and content, such as insults concerning sexual orientation, ethnic background, or appearance” (Oksanen et al., 2022, p. 542).	Oksanen et al. (2022)
Online Sexual Harassment	“The use of the Internet to sexually procure and/or intimidate an individual in some way” (Griffiths, 2000, p. 547).	Herovic et al. (2019)
Technology-Facilitated Violence and Abuse	“A host of harmful and disruptive behaviors that occur in online spaces and using digital communication technologies” (Gosse et al., 2024, p. 924).	Gosse et al. (2024)
Trolling	“Anti-social behavior aimed at inciting emotional reactions and derailing discussions in online communities” (Pasquier et al., 2024, p. 3).	Pasquier et al. (2024)
Trolling	“Experiencing one form of online abuse (posting of defamatory or false materials, racial abuse, sexual abuse, abuse on political grounds/beliefs, abuse on religious grounds/beliefs) and one form of online threatening behaviour (death threats, physical violence, rape, physical violence to friends and family, reputational damage, property damage” (Akhtar & Morrison, 2019, p. 323).	Akhtar and Morrison (2019)
Trolling	“The practice of behaving in a deceptive, destructive or disruptive manner in a social setting on the internet with no apparent instrumental purpose” (Buckels et al., 2014, p. 97).	Dineva and Breitsohl (2022)

**Table 3:** Distribution of articles across disciplines.

Field	Journal	Number of Articles
Computing and Information	Computers in Human Behavior	3
	Journal of Computational Social Science	1
	New Media & Society	1
	Information, Communication & Society	1
	Social Science Computer Review	1
	Internet Research	1
	Computer Supported Cooperative Work	1
	Social Media + Society	1
Communication	Western Journal of Communication	1
	International Journal of Sport Communication	1
	Communication & Sport	1
	Journal of Applied Communication Research	1
	Political Communication	1
	Media and Communication	1
Journalism	Journalism Practice	3
	Journalism Studies	1
Education	Higher Education	2
	Teaching and Teacher Education	1
Health and Social Care	British Journal of Social Work	1
	Healthcare	1
	Qualitative Social Work	1
Management	Organization	2
	International Journal of Manpower	1
Bullying	International Journal of Bullying Prevention	1
Law	The Journal of Legislative Studies	1
Psychiatry	Frontiers in Psychiatry	1
Science	Heliyon	1
Sexuality	Sexuality & Culture	1
Sport	Managing Sport and Leisure	1

## Data Availability

The data presented in this study are openly available in OSF project link: https://osf.io/tw54z/?view_only=2c374d394a434fbfad8022e3700420d6 (accessed on 8 April 2026). Anonymised Registry link: https://osf.io/4ahuj/?view_only=2ed450c05d07403d937b068b52a1a536 (accessed on 8 April 2026).

## Notes

1 Two studies examined samples of employed students. These studies were included in the review as the sample represented the working population.

2 Some studies used more than one data collection method.

## Supplementary Materials

The supporting information can be downloaded at: https://www.mdpi.com/article/10.3390/bs16050797/s1 (Tricco et al., 2018; Page et al., 2021).
