# Supplementary material for: Exploring Harassment Directed Towards Employees on Social Media: A Scoping Review
Source: Behav Sci (Basel). 2026 May 16;16(5):797. doi: 10.3390/bs16050797 (PMC13203256; doi:10.3390/bs16050797)
Supplement: Supplementary file 1 [file behavsci-16-00797-s001.zip › PRISMA2020 Flow Diagram.pdf]

PRISMA 2020 flow diagram for Exploring harassment directed towards employees on social media: A scoping review paper

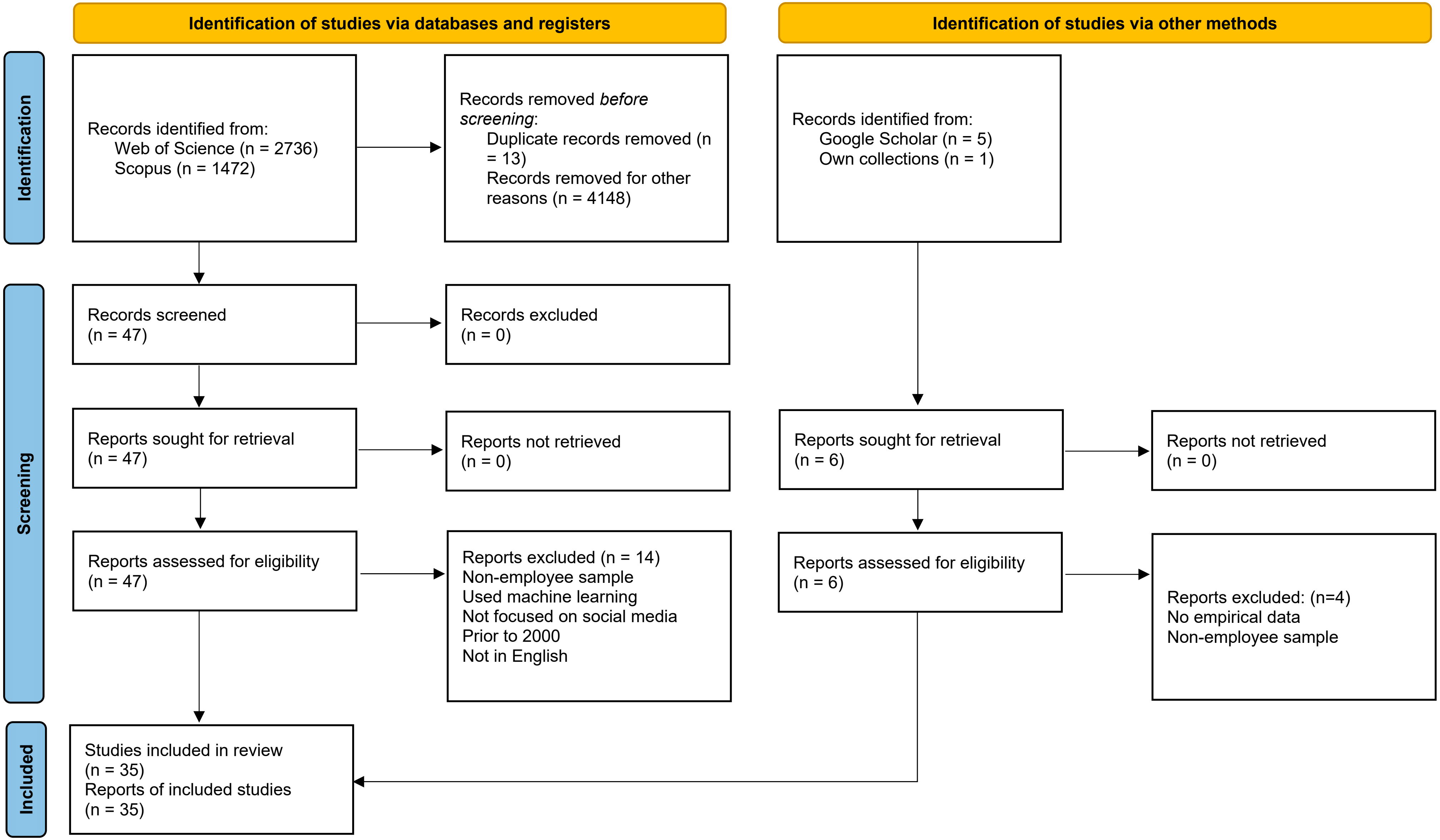

Source: Page MJ, et al. BMJ 2021;372:n71. doi: 10.1136/bmj.n71.
